# Comparison of Canal Transportation and Centering Ability of One-G, EdgeGlidePath, and Neolix: A MicroComputed Tomography Study of Curved Root Canals

**DOI:** 10.1155/2021/4898684

**Published:** 2021-11-09

**Authors:** Shiva Shojaeian, Niloofar Mortezapour, Fatemeh Soltaninejad, Nazanin Zargar, Babak Zandi, Yazdan Shantiaee, Amin Bidaki

**Affiliations:** ^1^Department of Endodontics, School of Dentistry, Shahid Beheshti University of Medical Sciences, Tehran, Iran; ^2^Student Research Committee, Babol University of Medical Sciences, Babol, Iran

## Abstract

**Aims:**

A glide path is created prior to root canal instrumentation by nickel-titanium (NiTi) rotary files to increase the efficiency and safety of cleaning and shaping. This study aimed to assess root canal transportation in use of different glide path files in curved canals.

**Materials and Methods:**

30 sound mesiobuccal root canals of mandibular molars with 20° to 40° curvature were selected and randomly assigned to 3 groups of EdgeGlidePath (EGP, EdgeEndo), One-G (Micro-Mega), and Neolix (Neoniti). The specimens were scanned before and after glide path creation by microcomputed tomography (micro-CT). The pre- and postoperative micro-CT scans were superimposed, and the degree of canal transportation and centering ratio were measured at 1, 3, 5, and 7 mm distances from the apical foramen. *Statistical Analysis*. The data were analyzed by two-way and one-way ANOVA.

**Results:**

The effects of distance from the apical foramen and instrument type and the interaction effect of the two were not significant on the centering ability of the files or canal transportation.

**Conclusion:**

EdgeGlidePath, One-G, and Neolix files fabricated from the conventional NiTi alloy or heat-treated M-Wire alloy showed similar performance regarding centering ability and canal transportation in glide path preparation in curved canals.

## 1. Introduction

Root canal instrumentation is an important step of root canal therapy during which the original shape and primary curvature of the root canals should be preserved; this is especially important in curved canals. For years, root canal preparation was performed by using stainless steel hand files, which would result in ledge formation, canal transportation, and perforation of the root canal system in many cases. Nickel-titanium (NiTi) rotary files were introduced to the market to minimize procedural errors during root canal preparation. NiTi files allow faster root canal instrumentation with a higher level of safety and decrease the frequency of procedural errors [1]. Procedural errors during root canal instrumentation weaken the root structure, hinder optimal cleaning, shaping, irrigation, and obturation of the root canal system, and can even lead to treatment failure [2].

Primary preparation of the root canal system is started by creating a glide path using stainless steel hand files or NiTi rotary instruments with a small diameter and taper [2, 3]. Glide path preparation refers to creation of a smooth tunnel from the root canal orifice to the apical foramen, which is an important step and increases the efficiency and safety of the cleaning and shaping of the root canal system [4–6]. Glide path creation decreases torsional stresses, increases the lifespan of the files, and decreases the rate of procedural errors during root canal preparation [4, 6, 7].

Use of hand files for the creation of a glide path has some advantages such as lower cost and better tactile sense [2, 3]. Nonetheless, use of these files fatigues the operator, prolongs the preparation time, increases the extrusion of debris through the apical foramen, and changes the original anatomy of the root canal system [7]. The rotary files used for the creation of a glide path allow faster root canal preparation with lower technical sensitivity and lower frequency of postoperative pain [2, 3].

Canal transportation refers to deviation from the original canal path during root canal preparation. Rotary files also have some problems in the creation of the glide path such as obstruction or transportation of the canal, lack of tactile sense, inadequate cleaning, and risk of file fracture [8]. Some manufacturers of rotary files use different file designs to minimize the risk of procedural errors and increase the speed of root canal instrumentation [9]. Evidence shows that NiTi files are superior to stainless steel files regarding shaping ability [10, 11].

Some methods are available to assess the centering ability of the files and the frequency of procedural errors such as root canal transportation during canal instrumentation, such as root sectioning at different levels from the apex [12], electron microscopic assessment, radiographic examination, and photographic examination. However, some of these techniques are invasive, and superimposition of specimens before and after treatment is difficult in some of them. By recent technological advances, use of noninvasive techniques such as microcomputed tomography (micro-CT) may provide precise information about the results of root canal instrumentation [13]. Limited studies have assessed root canal transportation by glide path rotary files using CT. This study aimed to assess root canal transportation by glide path rotary files in curved canals using micro-CT.

## 2. Materials and Methods

The study protocol was approved by the ethics committee of Shahid Beheshti University of Medical Sciences (IR.SBMU.DRC.REC.1398.173).

This in vitro, experimental study evaluated 30 mesiobuccal root canals of mandibular first and second molars with Vertucci's type IV mesial root and 20–40° curvature. The sample size was determined based on previous studies of this nature [5, 14, 15]. The teeth had intact root canals and had been extracted for reasons not related to this study. The selected teeth were inspected under a stereomicroscope at ×12 magnification to ensure absence of cracks and root resorption. Teeth with internal or external root resorption defects or calcification, cracked teeth, and those with merged mesial canals detected by radiography were excluded from the study. Next, the teeth underwent high-resolution cone-beam computed tomography using the NewTom VGi CBCT scanner (QR; SRL Co., Verona, Italy) with the exposure parameters of 110 kV, 9.5 mA, 1.0 mm voxel size, and 6 × 6 mm field of view. OnDemand3D software (Cybermed Inc., Irvine, CA, USA) was used for the assessment of different cone-beam computed tomography sections.

The root canals were randomly assigned to three groups for the use of EdgeGlidePath (EGP; EdgeEndo), One-G (Micro-Mega), and Neolix (Neoniti) files with 10 specimens in each group. The canal curvature was measured by using Schneider's technique [16]. All specimens were stored in 0.1% thymol solution during the experiment. The study groups and properties of the files are given in Table 1.

The crown and then distal root of the teeth were cut by a low-speed saw (Isomet 4000; Buehler Ltd., Lake Bluff, IL, USA) under water coolant, and the mesial root length was standardized to be 12 ± 1 mm from the apex. The working length was determined by observing the tip of a #8 K-file (Mani Inc., Tochigi, Japan) at the apex under ×10 magnification and subtracting 1 mm from this length. To simulate the periodontal ligament, the root surface was covered with one layer of aluminum foil, and the roots were then mounted in a plastic tube (55 mm diameter and 20 mm height) filled with acrylic resin (Kulzer GmbH, Leipziger, Hanau, Germany). Before complete setting of the acrylic resin, the aluminum foil was removed, and silicone impression material (GC Co., Tokyo, Japan) was used to fill the created space. The specimens were immediately mounted back in the block [2, 17].

A #10 K-file was then introduced into the canal in all teeth, and the glide path files were subsequently used with a VDW micromotor (VDW Silver Motor; VDW GmbH, Munich, Germany) according to the manufacturer's instructions. All specimens were prepared by the same operator, and each file was used only once. Any file fracture would be recorded. The root canals were irrigated with 2 mL of 1% sodium hypochlorite using a 27-gauge syringe after using each file. After each time of root canal irrigation, the patency of the canal was ensured by 1 mm using a #10 K-file. A final rinse was carried out with 3 mL of 17% EDTA.

All root canals underwent micro-CT (LOTUS-inVivo, Behin Negareh Co., Tehran, Iran) before and after using the glide path files. A total of 6 scans were obtained, all of them were evaluated, and the results were recorded. The entire process was automated. The LOTUS-inVivo scanner has a cone-shaped X-ray tube, a microfocus, and a flat-panel detector. To obtain maximum image quality, the X-ray tube voltage and amperage were 99 kV and 88 *µ*A, respectively, and the exposure time was 2 s. The entire scanning time was 60 min, and the slice thickness was 45 *µ*m. The entire process was controlled by LOTUS-inVivo-ACQ software program, and 3D data were reconstructed using the LOTUS-InVivo-REC algorithm [18, 19].

**Figure 1:**
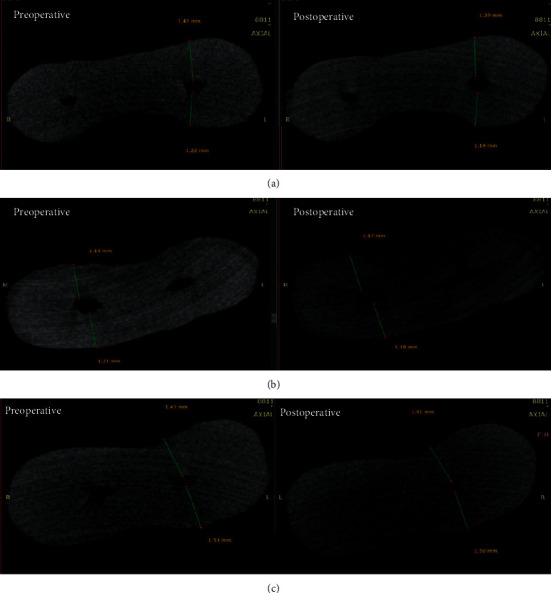
The pre- and postoperative images at 5 mm from the apical foramen: (a) One-G, (b) EdgeGlidePath, and (c) Neolix.

A reference marker was placed on the specimens mounted in acrylic resin, corresponding to the marker on the micro-CT scanner, enabling correct superimposition of pre- and postoperative images. By doing so, the degree of canal transportation and centering ratio were measured at 1, 3, 5, and 7 mm from the apical foramen (Figure 1). The pre- and postoperative images were reconstructed by software.

### 2.1. Canal Transportation

Canal transportation was calculated using the formula (*m*1 − *m*2) − (*d*1 − *d*2) and recorded. If the obtained value was zero, it indicated no canal transportation; if the obtained value was positive, it would indicate deviation towards the mesial border, and if it was negative, it would indicate deviation towards the distal border [20].

### 2.2. Centering Ratio

The centering ratio was calculated using the formula (*m*1 − *m*2)/(*d*1 − *d*2). If the obtained value was 1, it would indicate excellent centering ability, and if it was 0, it would indicate poor centering ability of the file [20].

(1) *m*1: minimum distance between the mesial border of the canal and mesial border of the root before instrumentation
(2) *m*2: minimum distance between the mesial border of the canal and mesial border of the root after instrumentation
(3) *d*1: minimum distance between the distal border of the canal and distal border of the root before instrumentation
(4) *d*2: minimum distance between the distal border of the canal and distal border of the root after instrumentation

### 2.3. Statistical Analysis

Data were analyzed using SPSS version 25.0. The measures of central dispersion (mean and standard deviation) were calculated and reported for canal transportation and centering ability of different glide path files at different levels from the apical foramen. The parametric two-way ANOVA was applied to analyze the effects of file type, distance from the apical foramen, and their interaction effect on canal transportation and centering ability. Also, different glide path files were compared with each other regarding canal transportation and centering ability using one-way ANOVA. Type I error was considered to be 0.05 (*α* = 0.05).

## 3. Results

Table 2 presents the mean canal transportation and centering ratio of One-G, EdgeGlidePath, and Neolix files at 1, 3, 5, and 7 mm from the apex. According to two-way ANOVA, the effects of distance from the apical foramen (*P*=0.099), file type (*P*=0.198), and the interaction effect of the two (*P*=0.753) on canal transportation were not significant. Also, one-way ANOVA revealed no significant difference in canal transportation between the files at 7 mm (*P*=0.86), 5 mm (*P*=0.16), 3 mm (*P*=0.72), and 1 mm (*P*=0.65) from the apical foramen.

According to two-way ANOVA, the effects of distance from the apical foramen (*P*=0.481), file type (*P*=0.295), and the interaction effect of the two (*P*=0.224) on centering ratio were not significant. Also, one-way ANOVA revealed no significant difference in centering ratio between the files at 7 mm (*P*=0.133), 5 mm (*P*=0.086), 3 mm (*P*=0.5), and 1 mm (*P*=0.808) from the apical foramen.

## 4. Discussion

Creation of a glide path is imperative for optimal function of NiTi files [21]. Mechanical creation of a glide path is a fundamental step for the reduction of the effects of torsional stress along the root canal, the screwing effects of rotary instruments, and the risk of instrument fracture [22]. Also, mechanical creation of a glide path can expedite the process of instrumentation and decrease the frequency and severity of postendodontic pain [23]. Some methods are available to assess the centering ability of the files and the frequency of procedural errors such as root canal transportation during canal instrumentation, such as root sectioning at different levels from the apex [13], electron microscopic assessment, radiographic examination, and photographic examination. However, some of these techniques are invasive, and superimposition of specimens before and after treatment is difficult in some of them. By recent technological advances, use of noninvasive techniques such as microcomputed tomography (micro-CT) may provide precise information about the results of root canal instrumentation [14]. Limited studies have assessed root canal transportation by glide path rotary files using CT. This study aimed to assess root canal transportation by glide path rotary files in curved canals using micro-CT.

The results showed that root canal transportation occurred in use of all glide path files and at all levels from the apical foramen. Minimum canal transportation was recorded at 1 mm from the apex in use of the EdgeGlidePath file by 0.002 mm, while maximum canal transportation occurred at 5 mm from the apex by 0.07 mm in use of the EdgeGlidePath file. This finding may be due to the progressive taper of the EdgeGlidePath file, compared with the other two systems, although the difference in canal transportation was not significant among the three systems.

In the present study, the centering ability of all three files was the same at all 4 levels from the apical foramen. This result was in agreement with the findings of other studies that assessed glide path creation by the use of different NiTi rotary files [22, 24]. Cesaitiene et al. assessed the canal transportation and centering ability of different techniques of glide path preparation by the use of rotary files in curved root canals. They demonstrated that all rotary systems had a similar performance in this respect [25]. They used PathFile and ProGlider files in their study, which are different from the file systems used in the present study. However, the technique of the assessment of the centering ability of the files was micro-CT in both studies. Vyver et al. assessed the centering ratio and canal transportation following the use of K-files, ProGlider, and One-G for the creation of a glide path in curved canals. They reported that One-G and ProGlider had higher centering ratio than K-files in all areas [26]. In the present study, the centering ability of all tested files was almost the same; however, K-files were not evaluated in the present study. Aydin et al. evaluated the canal transportation and centering ability of ProGlider, WaveOne Gold Glider, and R-Pilot using micro-CT and did not find significant differences in canal transportation in the apical third. Their results were in agreement with our findings [2] despite the use of different systems.

All root canal preparation instruments and techniques tend to change the original canal path and cause transportation to some extent [12] because all endodontic files are fabricated from a straight hard metal wire. Thus, stresses do not have a uniform distribution at the contact area of the instrument with the canal. Resultantly, the instrument wants to straighten up in the canal, causing additional forces to the external surface of the canal curvature (the concave part), which results in canal transportation [27].

Also, there is a possibility that the changes caused in the process of glide path preparation are further aggravated in the process of root canal shaping because the longitudinal axis of the curved canals changes in this process, the degree of curvature decreases, and the original curvature of the canal becomes rather straight [26].

The shaping efficacy of endodontic instruments can be assessed by 2D or 3D imaging modalities such as micro-CT. In 2D radiography, digital radiographic images are superimposed in two vertical directions, and a software program is used to calculate the degree of canal transportation with or without superimposition of images [28].

Cross-sectioning of the canals is another technique that enables direct observation of the root canal shape. However, the original canal path prior to instrumentation cannot be studied in this technique [28]. Micro-CT is increasingly used for the assessment of the efficacy of new endodontic instruments due to its noninvasiveness [29, 30]. In this technique, the root canal morphology is studied preoperatively and prior to probing of the canal with small hand files. Thus, the root canal anatomy is studied in its original intact form [7]. Peters et al. reported that studies on the effects of root canal preparation on the root canal anatomy should include the details of the root canal geometry prior to instrumentation in their results because the variations in root canal geometry have a greater effect on the changes caused by root canal preparation than the technique itself [31].

In the present study, the mesiobuccal root canals of mandibular molar teeth were evaluated because these canals often have significant curvature and are considerably narrow [7]. These properties can significantly increase the level of difficulty of root canal preparation [7, 32].

In the present study, canal transportation and centering ratio were evaluated at 4 levels from the apex (at 1, 3, 5, and 7 mm distances from the apical foramen) [33]. These distances represented the foraminal, apical, middle, and coronal third of the root, where root curvature often leads to procedural errors [34].

This study was conducted under in vitro conditions, and high standard deviation values due to the use of natural teeth were a limitation of this study. Therefore, the results should be generalized to the clinical setting with caution. Future clinical trials are required to confirm the current findings.

## 5. Conclusion

The results of the current study on canal transportation and centering ratio of different glide path files in curved canals revealed an insignificant effect of distance from the apical foramen and file type or their interaction on canal transportation and centering ratio. The difference in centering ability or canal transportation of the files at 1, 3, 5, and 7 mm from the apex was not significant either. EdgeGlidePath, One-G, and Neolix files fabricated from the conventional NiTi or heat-treated M-Wire alloy showed similar performance with regard to canal transportation and centering ability in the process of glide path preparation in curved canals in vitro.

## Figures and Tables

**Table 1:** Properties of the files.

Name	Size	Speed (rpm)	Torque (N/cm)	Alloy material
Neolix	#15, 3%	250	1.5	Treated NiTi
EdgeGlidePath	#16, progressive	350	2	Heat-treated NiTi
One-G	#14, 3%	350	1.2	Conventional NiTi

**Table 2:** Mean canal transportation and centering ratio of One-G, EdgeGlidePath, and Neolix files at 1, 3, 5, and 7 mm from the apex.

File	Parameter	1 mm (mean ± SD)	3 mm (mean ± SD)	5 mm (mean ± SD)	7 mm (mean ± SD)
One-G	Transportation	0.014 ± 0.066^a^	0.007 ± 0.047^a^	0.029 ± 0.069^a^	0.017 ± 0.044^a^
	Centering ratio	0.461 ± 0.287^a^	0.614 ± 0.294^a^	0.443 ± 0.212^a^	0.673 ± 0.174^a^

EdgeGlidePath	Transportation	0.002 ± 0.032^a^	0.011 ± 0.06^a^	0.07 ± 0.197^a^	0.019 ± 0.055^a^
	Centering ratio	0.459 ± 0.352^a^	0.493 ± 0.229^a^	0.643 ± 0.305^a^	0.639 ± 0.349^a^

Neolix	Transportation	0.019 ± 0.052^a^	0.004 ± 0.051^a^	0.03 ± 0.086^a^	0.03 ± 0.068^a^
	Centering ratio	0.537 ± 0.269^a^	0.487 ± 0.283^a^	0.381 ± 0.265^a^	0.434 ± 0.281^a^

Different superscripted letters indicate a significant difference between the groups (*P* < 0.05).

## Data Availability

The datasets analyzed during the current study are available from the corresponding author upon reasonable request.
